# Recurrence Rate and Relevant Associated Factors of Stroke among Patients with Small Artery Occlusion in Northern China

**DOI:** 10.1038/s41598-019-39207-0

**Published:** 2019-02-26

**Authors:** Ying Zhang, Yalin Guan, Yajing Zhang, Shuling Liu, Man Zheng, Min Wang, Wenhua Su, Hao Wu

**Affiliations:** 1 Department of Neurology, Tianjin Huanhu Hospital, Tianjin, 300350 China; 2 Tianjin Key Laboratory of Cerebral Vascular and Neurodegenerative Disease, Tianjin, 300350 China; 3 Department of Cardiology, Dongying People’s Hospital, Shandong Province, Dongying 257091 China; 4 Department of Neurology, Dongying People’s Hospital, Shandong Province, Dongying 257091 China

## Abstract

Small artery occlusion (SAO) is responsible for 31.3% of all ischemic strokes in China. However, reports regarding the recurrence rate of SAO in China are rare. We aimed to assess the recurrence rate and factors associated with SAO in China. All consecutive patients with SAO hospitalized at Tianjin Huanhu Hospital from 2005 to 2014 were recruited. We assessed the association between stroke subtype, severity, and disease history with recurrence at 3, 12, and 36 months of onset using multivariate logistic regression analysis. A total of 2,524 SAO patients were included in this study, including 1696 (67.2%) men and 828 (32.8%) women. The recurrence rates were 3.1% at 3 months, 12.7% at 12 months, and 36.5% at 36 months. Compared with women, men had a higher risk of recurrence at 3 months after SAO (P = 0.003). Old age and severity of stroke were also associated with a higher risk of recurrence (P < 0.05). Patients with an elevated C-reactive protein had a higher risk of recurrence at 12 months (P = 0.003). On the other hand, the risk of recurrence at 12 months was 39% lower in patients who consumed alcohol than in those who did not (P = 0.037). Hypertension, atrial fibrillation, and obesity were independent risk factors of recurrence at 36 months. These findings suggest that modification of risk factors in patients with SAO, particularly men, is essential for reducing the rate of recurrence and the overall burden of stroke in China.

## Introduction

Stroke has become the second most common cause of death and the leading cause of disability worldwide^1^. In China, 21.6% of deaths among men and 20.8% of deaths among women are attributed to stroke^2^. Moreover, stroke has become the leading cause of death in rural areas and the third most common cause of death in urban areas in China^3^. While the incidence of stroke has declined in developed countries over the past several decades^4,5^, it has increased in developing countries, particularly in China^6–8^. Furthermore, the total incidence of stroke is expected to increase considerably over the next two decades^9^.

Stroke is a complex disease caused by multiple potential etiologies. It is important to identify the underlying causes and the subtype of stroke in order to determine the most appropriate treatment and to accurately predict patient outcomes^10^. Small artery occlusion (SAO) is a distinct type of ischemic stroke according to the Trial of ORG10172 in Acute Stroke Treatment (TOAST) classification. The age-standardized incidence rate of SAO has been reported to be 25.8 per 100,000 persons (95% confidence interval, 21.5, 30.9) in Europe^11^. SAO accounts for approximately 20% of all ischemic strokes in Europe^12^, but up to 31.3% of cases in China^13^.

Previous studies have demonstrated that disability, mortality, and recurrence rates after ischemic stroke are associated with stroke subtype^11,14^. However, there is limited evidence on the recurrence rates of SAO in China. Thus, it is crucial to assess the recurrence rate and the relevant patient factors associated with SAO in order to manage risk and improve secondary prevention, particularly since SAO is associated with longer survival and lower severity compared with other stroke subtypes^15^.

In this study, we identified the recurrence rates and relevant risk factors at 3, 12, and 36 months after stroke among patients with SAO at a single center in China.

## Results

### Description of the demographical and clinical features among SAO patients

**Figure 1:**
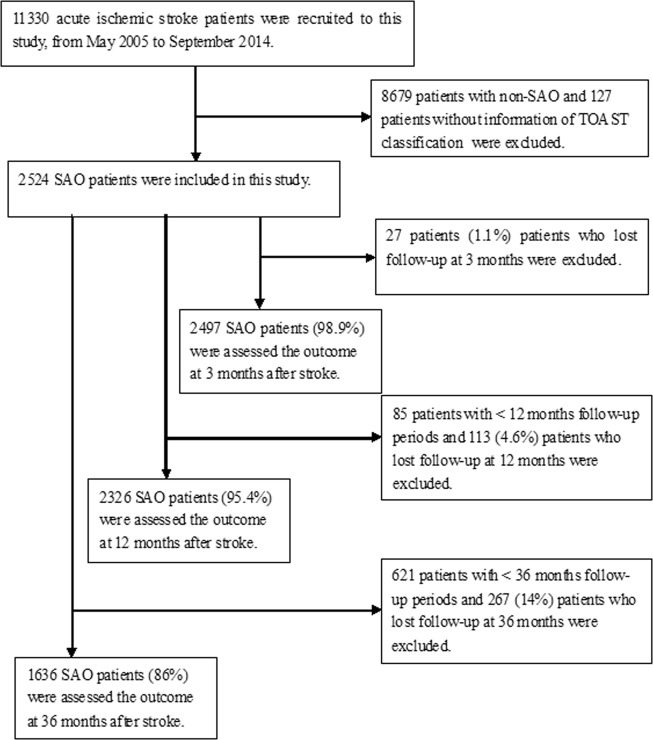
Flow chat of patients’ selection. A total of 11,330 patients with acute ischemic stroke were recruited during the study period. After excluding 8679 patients with non-SAO strokes and 127 without TOAST classification data, the final analysis included 2524 patients with SAO. Follow-up data for the assessment of recurrence was available for 2497 SAO patients (98.9%) at 3 months, 2326 (95.4%) at 12 months, and 1636 (86%) at 36 months.

A total of 11,330 patients with acute ischemic stroke were recruited during the study period. After excluding 8679 patients with non-SAO strokes and 127 without TOAST classification data, the final analysis included 2524 patients with SAO. Follow-up data for the assessment of recurrence was available for 2497 SAO patients (98.9%) at 3 months, 2326 (95.4%) at 12 months, and 1636 (86%) at 36 months (Fig. 1).

Men comprised 67.2% (n = 1,696) of the SAO cohort while women accounted for 32.8% (n = 828), with an average age of 62.71 years. Most patients were between 50 and 64 years (55.9%), and mild stroke was the most common (82.7%). The prevalence of hypertension, diabetes mellitus, atrial fibrillation (AF), artery stenosis, obesity, current smoking, and alcohol consumption was 75.9%, 32.4%, 2.3%, 13.9%, 11.5%, 37.8%, and 18.8%, respectively. Moreover, the average levels of fasting plasma glucose, total cholesterol, triglycerides, high-density lipoprotein cholesterol, low-density lipoprotein cholesterol, high-sensitivity C-reactive protein (Hs-CRP), and homocysteine (HCY) were 6.44 mmol/L, 4.92 mmol/L, 1.70 mmol/L, 1.06 mmol/L, 3.00 mmol/L, 6.23 mmol/L, and 15.89 mmol/L, respectively (Table 1).

**Table 1:** Descriptive characteristics in clinical features and risk factors among patients with SAO by gender groups.

Characteristics	value	P
Gender		
Men	1696 (67.2)	
Women	828 (32.8)	
Total	2524 (100)	
Age, years, means (SD)	62.71 (11.50)	
Age group, n (%)		<0.001
<50 years	304 (12.0)	
50 years~	700 (27.7)	
60 years~	711 (28.2)	
70 years~	594 (23.5)	
≥80 years	215 (8.5)	
Stroke severity		0.003
Mild	2083 (82.7)	
Moderate	375 (14.9)	
Severe	62 (2.5)	
Risk factors, n (%)		
Hypertension	1916 (75.9)	
Diabetes	817 (32.4)	
Atrial fibrillation	59 (2.3)	
Artery stenosis	351 (13.9)	
Obesity	291 (11.5)	
Current smoking	953 (37.8)	
Alcohol drinking	474 (18.8)	
Neurological function^*^		
NIHSS	4 (5)	
BI	75 (45)	
mRS	2 (3)	
Laboratory testing		
FPG, mmol/L, means (SD)	6.44 (2.72)	
TC, mmol/L, means (SD)	4.92 (1.00)	
TG, mmol/L, means (SD)	1.70 (1.33)	
HDL-C, mmol/L, means (SD)	1.06 (0.27)	
LDL-C, mmol/L, means (SD)	3.00 (0.81)	
Hs-CRP, mmol/L, means (SD)	6.23 (19.90)	
HCY, mmol/L, means (SD)	15.89 (11.60)	

^*^Indicated that data were presented as median with interquartile range.

### Risk factors for recurrence on univariate analysis

The overall 3-month recurrence rate was 3.1%, with 3.8% in men and 1.7% in women. The corresponding recurrence rates at 12 and 36 months after SAO were 12.7%, 12.4%, and 13.2%; and 36.5%, 35.2%, and 34.4%, respectively. Recurrence was correlated with older age (P = 0.036 for 3 months and < 0.001 for 12 months and 36 months) and stroke severity (P = 0.008 for 3 months, P < 0.001 for 12 months and 36 months). Moreover, sex was associated with recurrence at 3 months after stroke; diabetes, current smoking, alcohol consumption, and Hs-CRP levels at admission were associated with recurrence at 12 months; and hypertension, diabetes, AF, obesity, current smoking, and Hs-CRP levels at admission were associated with recurrence at 36 months after SAO (all P < 0.05; Tables 2 and 3).

**Table 2:** Risk factors of recurrence among patients with SAO at 3, 12, and 36 months after stroke.

Outcomes	3 months		12 months		36 months	
	n (%)	P	n (%)	P	n (%)	P
Gender, n (%)	76 (3.1)	0.006	284 (12.7)	0.612	546 (36.5)	0.129
Men	62 (3.8)		188 (12.4)		358 (35.2)	
Women	14 (1.7)		96 (13.2)		188 (39.2)	
Age group		0.036		<0.001		<0.001
<50 years	7 (2.3)		23 (8.1)		52 (29.5)	
50 years~	18 (2.6)		76 (11.8)		126 (27.2)	
60 years~	21 (3.0)		65 (22.9)		154 (38.8)	
70 years~	17 (2.9)		86 (30.3)		179 (48.5)	
≥80 years	13 (6.6)		34 (12.0)		35 (38.9)	
Stroke severity		0.008		<0.001		<0.001
Mild	56 (2.7)		218 (11.6)		416 (33.2)	
Moderate	16 (4.5)		56 (18.1)		113 (52.8)	
Severe	4 (7.8)		10 (24.4)		15 (62.5)	
OCSP Classification		0.216		0.892		0.045
PACI	24 (2.5)		107 (12.3)		226 (40.4)	
TAPI	2 (4.8)		5 (14.3)		9 (40.9)	
LACI	24 (3.5)		87 (13.6)		168 (32.5)	
POCI	26 (3.4)		85 (12.2)		143 (36.0)	
Hypertension		0.753		0.499		0.018
Yes	59 (3.2)		220 (12.9)		441 (38.1)	
No	17 (2.9)		64 (11.8)		105 (31.1)	
Diabetes		0.921		0.043		0.013
Yes	24 (3.0)		106 (14.7)		195 (41.1)	
No	52 (3.1)		178 (11.7)		351 (34.4)	
Atrial fibrillation		0.322		0.515		0.001
Yes	3 (5.4)		7 (15.9)		17 (68.0)	
No	73 (3.0)		277 (12.6)		529 (36.0)	
Artery stenosis		0.847		0.184		0.610
Yes	10 (2.9)		45 (15.1)		74 (38.1)	
No	66 (3.1)		239 (12.3)		472 (36.3)	
Obesity		0.536		0.753		0.003
Yes	7 (2.5)		30 (12.0)		78 (47.0)	
No	69 (3.2)		254 (12.8)		468 (35.2)	
Current smoking		0.794		0.002		<0.001
Yes	30 (3.2)		85 (9.9)		170 (29.9)	
No	46 (3.0)		199 (14.4)		376 (40.5)	
Alcohol drinking		0.056		0.001		0.060
Yes	8 (1.7)		34 (7.7)		92 (31.7)	
No	68 (3.4)		250 (13.9)		454 (37.6)

**Table 3:** Differences in laboratory testing between recurrence and non-recurrence patients with SAO at 3, 12, and 36 months after stroke.

Laboratory testing	3 months		12 months		36 months	
	means (SD)	P	means (SD)	P	means (SD)	P
FPG, mmol/L		0.927		0.667		0.087
Recurrence	6.43 (3.20)		6.42 (2.82)		6.38 (2.79)	
Non-recurrence	6.39 (2.69)		6.34 (2.69)		6.12 (2.28)	
TC, mmol/L		0.487		0.943		0.864
Recurrence	4.99 (1.21)		4.88 (1.11)		4.88 (1.10)	
Non-recurrence	4.89 (0.99)		4.89 (0.99)		4.87 (0.96)	
TG, mmol/L		0.425		0.809		0.250
Recurrence	1.56 (0.73)		1.67 (0.86)		1.63 (0.97)	
Non-recurrence	1.68 (1.26)		1.69 (1.34)		1.71 (1.53)	
HDL-C, mmol/L		0.555		0.131		0.225
Recurrence	1.04 (0.34)		1.03 (0.30)		1.05 (0.29)	
Non-recurrence	1.08 (0.53)		1.09 (0.56)		1.07 (0.28)	
LDL-C, mmol/L		0.183		0.127		0.100
Recurrence	3.15 (1.03)		3.06 (0.87)		3.02 (0.80)	
Non-recurrence	2.99 (0.79)		2.98 (0.77)		2.95 (0.76)	
Hs-CRP, mmol/L		0.917		0.002		0.007
Recurrence	6.26 (6.98)		10.70 (28.54)		8.37 (29.17)	
Non-recurrence	6.00 (19.87)		4.90 (17.14)		4.55 (15.49)	
HCY, mmol/L		0.181		0.146		0.890
Recurrence	17.67 (13.28)		16.69 (12.63)		15.83 (11.36)	
Non-recurrence	15.80 (11.55)		15.61 (11.10)		15.75 (10.83)	

### Determinants of recurrence on multivariate analysis

Compared to women, men were more likely to experience a recurrence at 3 months after SAO (relative risk [95% confidence interval], 2.50 [1.38 to 4.53; P = 0.003]). Old age was a common risk factor for recurrence at 3, 12, and 36 months after SAO. Patients ≥80 years had a 2.07 and 1.12-fold increased risk of recurrence than those <50 years at 3 and 12 months, respectively (P < 0.05). Meanwhile, there was a 77% higher risk of recurrence at 36 months after SAO in patients between 70 and 79 years (P = 0.007). In comparison to patients with mild stroke, there was an increased risk of recurrence at 12 months in those with moderate stroke, and at 36 months in those with moderate and severe strokes. Moreover, the risk of recurrence at 12 months after SAO was 39% lower in patients who drank alcohol compared to those who did not (P = 0.037). The risk of recurrence increased in accordance with CRP level elevation (P = 0.003). Hypertension, AF, and obesity were independent risk factors of recurrence at 36 months after SAO (relative risk [95% confidence interval], 1.38 [1.04, 1.84; P = 0.027], 3.09 [1.28, 7.47; P = 0.012], and 1.62 [1.13, 2.31; P = 0.008], respectively; Table 4).

**Table 4:** Determinants of recurrence at 3, 12, and 36 months after SAO.

Characteristics	References	3 months		12 months		36 months	
		OR (95%CI)	P	OR (95%CI)	P	OR (95%CI)	P
Men	Women	2.50 (1.38, 4.53)	0.003	—	—	—	—
Age groups	<50 Years						
50 years~		1.21 (0.50, 2.93)	0.674	1.17 (0.71, 1.94)	0.543	0.82 (0.55, 1.23)	0.334
60 years~		1.40 (0.59, 3.35)	0.445	0.98 (0.58, 1.65)	0.934	1.25 (0.83, 1.87)	0.290
70 years~		1.41 (0.57, 3.46)	0.457	1.48 (0.89, 2.48)	0.133	1.77 (1.17, 2.69)	0.007
≥80 years		3.07 (1.18, 7.99)	0.021	2.12 (1.16, 3.88)	0.015	1.31 (0.74, 2.33)	0.354
Stroke severity	Mild						
Moderate		1.68 (0.94, 2.98)	0.078	1.43 (1.01, 2.34)	0.044	1.82 (1.32, 2.50)	<0.001
Severe		2.62 (0.88, 7.83)	0.084	1.42 (0.62, 3.24)	0.404	2.55 (1.03, 6.33)	0.043
Hypertension	No	—	—	—	—	1.38 (1.04, 1.84)	0.027
Diabetes	No	—	—	1.12 (0.84, 1.48)	0.448	1.26 (0.98, 1.61)	0.068
AF	No	—	—	—	—	3.09 (1.28, 7.47)	0.012
Obesity	No	—	—	—	—	1.62 (1.13, 2.31)	0.008
Current smoking	Never	—	—	0.88 (0.63, 1.23)	0.448	0.86 (0.67, 1.10)	0.231
Alcohol drinking	Never	—	—	0.61 (0.39, 0.97)	0.037	—	—
Hs-CRP		—	—	1.008 (1.003, 1.013)	0.003	1.005 (0.999, 1.011)	0.109

## Discussion

In the present study, we assessed the recurrence rates and their associated factors among patients with SAO at 3, 12, and 36 months after stroke. To our knowledge, this is the first study to report the recurrence rates and determinants of SAO in China. The recurrence rates were 3.1% at 3 months, 12.7% at 12 months, and 36.5% at 36 months after SAO. Male sex and older age (≥80 years) were independent risk factors for recurrence at 3 months after SAO. Older age (≥80 years), moderate stroke, and elevated CRP level were independent risk factors of recurrence at 12 months after SAO, but alcohol drinking was negatively associated with recurrence. Older age (70–79 years), moderate/severe stroke, hypertension, AF, and obesity were determinants of recurrence at 36 months after SAO. The Oxford Vascular Study had shown that the risk of recurrence varied between stroke subtypes and the 3-month rate for SAO was 6.2%^14^.

The outcomes and recommended therapeutic interventions differ according to the stroke subtype^14,16^. Although SAO is typically associated with more favorable outcomes, the expected long-term survival following SAO can increase the risk of adverse events, particularly recurrence.

Previous studies have demonstrated poor stroke outcomes among elderly patients^17–21^. However, reports of outcomes among patients with SAO have been limited and controversial. The previous studies demonstrated favorable short-term outcomes among SAO patients^22,23^, but the unfavorable long-term outcomes among elderly SAO patients^22,24^. Moreover, young patients with SAO have low 5-year mortality rates^25^. In this study, the short and long-term risk of recurrence was higher among elderly patients.

Several studies have reported that the National Institutes of Health Stroke Scale (NIHSS) score was highly related to stroke outcome^18,26,27^. Ischemic stroke subtypes according to the TOAST criteria were correlated with short- and long-term recurrence after stroke^14,28^. Age, NIHSS score on admission, and female sex were independent risk factors for poor outcome (defined as a modified Rankin Scale [mRS] score >2 or death at 3-month follow-up)^29^. A higher rate of recurrence was observed in men with SAO than in women^30^. In the present study, we found that stroke severity according to the NIHSS score on admission was significantly associated with long-term recurrence (12 and 36 months). However, we found a higher risk of recurrence at 3 months in male patients. Moreover, hypertension, AF, and obesity increased the risk of recurrence in patients at 36 months. Poor control of risk factors (including hypertension, AF, and obesity) may contribute to recurrence in SAO patients, particularly in men.

CRP is currently used for evaluating pathological inflammation and has been extensively studied in relation to the progression of atherosclerosis^31^. The level of Hs-CRP at admission predicts future recurrence in patients with acute ischemic stroke^32,33^. Moreover, a recent study revealed that higher CRP levels were associated with subclinical markers of cerebral small vessel disease and neurodegeneration^34^. In the present study, we found that elevated levels of Hs-CRP increased the risk of recurrence of SAO at 12 months. Several mechanisms may explain this finding. First, elevated CRP levels are involved in vessel occlusion, altered cerebral autoregulation, increased vascular permeability, and are a marker of arteriolosclerosis^35^. Second, elevated CRP is associated with large-vessel atherosclerosis, which affects the microvascular endothelium of cerebral small vessels by reducing cerebral blood flow and producing inflammatory mediators and free radicals^36^. Finally, CRP is associated with endothelial cell activation, which plays a key role in small- and large-vessel disease^37^.

Previously reports on the effects of alcohol consumption on outcome after stroke have been controversial. Excessive alcohol consumption showed no association with functional outcome after stroke^38,39^. The North East Melbourne Stroke Incidence Study found that pre-stroke alcohol consumption did not independently predict disability at 2 years after stroke on multivariate analysis^40^. The Copenhagen Stroke Study demonstrated that daily alcohol consumption was not an independent predictor of good outcomes on multivariate analysis^41^. A population-based study indicated that light-to-moderate alcohol consumption was not associated with reduced stroke risk^42^, while heavier consumption increased the risk of stroke or death^42,43^. In contrast to these previous studies, we found that alcohol consumption was associated with a low risk of recurrence at 12 months after SAO. Differences in the study participants (general population or patients with stroke), alcohol consumption style (categorized by qualitative or quantitative factors), and study endpoints (risk of developing stroke or prognosis after stroke) may partly explain this disparity. Moreover, differences between the life styles of Western and Eastern populations may have contributed to inversion of effects, especially in terms of alcohol consumption. Some studies have reported that excessive alcohol consumption increases the relative risk of stroke, while light consumption may be protective against ischemic stroke^44–46^.

There are several limitations to this study. First, this study was conducted at a single hospital and thus represents a limited population. Moreover, Tianjin Huanhu Hospital is a specialized neurology and neurosurgery hospital in Tianjin, China. The patients treated here are usually in a more critical condition than those at other hospitals and this may result in selective bias. Additionally, we assessed the association of alcohol consumption with the risk of recurrence after SAO using the qualitative categorization (no drinking versus current drinking) those ever alcohol drinkers was involved in no drinking group. Moreover, there was no quantitative analysis of alcohol consumption per day or week. Further quantitative evaluation of the impact of alcohol consumption on the prognosis after SAO is urgently needed. Finally, this study may have excluded some patients with severe stroke and those who died before hospital admission, which could have resulted in an unfair assessment.

In conclusion, we observed a lower rate of recurrence at 3 months after SAO, but a 4- and 12-fold increase in risk at 12 and 36 months, respectively. Age, sex, stroke severity, hypertension, AF, obesity, and elevated CRP level were associated with an increased risk of recurrence, while alcohol consumption decreased this risk. Poor management and uncontrolled risk factors might contribute to the high risk of recurrence among SAO patients. These findings suggest that improving risk factor control in patients with SAO, particularly among male patients, is critical to reducing the recurrence rate and alleviating the overall stroke burden in China.

## Methods

All patients in this study were recruited from the stroke unit at Tianjin Huanhu Hospital, China between May 2005 and September 2014.The procedure used to establish the stroke registry has been described previously^47–49^. In brief, all consecutive patients with acute ischemic stroke were entered into the registry system, and detailed information, including clinical features and outcomes, were collected.

The ethics committee of Tianjin Huanhu Hospital approved the study, and written informed consent was obtained from all patients or their next-of-kin.

Stroke patients were identified according to the World Health Organization’s criteria, and all cases of stroke were confirmed using neuroimaging^50^. All acute ischemic stroke patients were categorized as either of five etiological subtypes according to their medication history, location of ischemic zone, and vessels status (TOAST classification). First, computed tomography (CT) or magnetic resonance imaging (MRI) was performed to ascertain the location and diameter of the ischemic focus in all patients. Patients with penetrating artery or lacunar infarctions, and a focus diameter of <1.5 cm on CT or MRI were classified SAO^51^. All patients subsequently underwent carotid ultrasonography for exclusion of large artery stenosis, and echocardiography for exclusion of cardiac emboli. Some patients with abnormal blood flow velocities, multiple carotid plaques, or severe artery stenosis underwent additional CT or magnetic resonance angiography.

Stroke severity was categorized into three groups according to NIHSS scores: mild (NIHSS score: ≤7), moderate (NIHSS score: 8–16), and severe (NIHSS score: ≥17)^52^. Patients were considered to have been using oral anticoagulants if they were receiving oral anticoagulants prior to stroke onset. Stroke risk factors, such as previous medical history (including hypertension, diabetes mellitus, AF, hyperlipidemia, and obesity) and life style (including smoking and alcohol consumption) were recorded on admission. The presence of hypertension, diabetes mellitus, and hyperlipidemia were defined according to self-reporting of disease history or use of antihypertensives, antidiabetics, or lipid-lowering medication, respectively. AF was defined as a history of AF, confirmed through at least one electrocardiogram, or the presence of arrhythmia during hospitalization. Obesity was defined as a body mass index ≥30 kg/m^2^. The NIHSS score and Barthel Index were evaluated on admission and at discharge. The mRS score was assessed on admission; at discharge; and at 3, 12, and 36 months after stroke. Moreover, TC, (TG), HDL-C, LDL-C, FPG, Hs-CRP, and HCY levels on admission were recorded.

Recurrence was defined as all new-onset vascular events, including stroke, myocardial infarction, and venous thrombosis after 1 month of stroke onset. Follow-up was conducted according to a predetermined procedure: trained neurologists assessed patients in the outpatient department at 3, 12, 24, and 36 months after stroke. All patients were followed-up by face-to-face interview, but those reexamined at the local hospital were followed-up by telephone.

The metric variables are presented as means with standard deviations for age as well as TC, TG, HDL-C, LDL-C, Hs-CRP, and HCY levels. NIHSS score, Barthel Index, and mRS score are presented as medians with ranges. Differences in metric variables between groups were compared using Student’s t-test or the Mann-Whitney U test. Categorized variables, including sex, stroke severity, stroke risk factors, and recurrence rate during follow-up, are presented as number of cases (percentages). Differences in risk factors were compared between groups using the Chi-squared test. Multivariate analysis of determinants of recurrence (as a dichotomous variable: yes or no) was assessed using logistic regression after adjusting for covariables, which included age (<75 years, ≥75 years), sex (male, female), stroke severity (mild, moderate, severe), medical history (as a dichotomous variable: yes or no), risk factors (as a dichotomous variable: yes or no), and laboratory measurements (as a continuous variable). The results are presented as adjusted relative risk with 95% confidence intervals. All statistical analyses were performed using SPSS version 19.0 (SPSS Inc., Chicago, IL), and a two-tailed P value < 0.05 indicated statistical significance.
